# Metabolic engineering of *Corynebacterium crenatium* for enhancing production of higher alcohols

**DOI:** 10.1038/srep39543

**Published:** 2016-12-20

**Authors:** Haifeng Su, Jiafu Lin, GuangWei Wang

**Affiliations:** 1 Chongqing Institute of Green and Interligent Technology, Chinese Academy of Science. 266, Fangzheng Avenue, Shuitu High-tech Park, Beibei, Chongqing 400714, China; 2 Antibiotics Research and Re-evaluation Key Laboratory of Sichuan Province, Sichuan Industrial Institute of Antibiotics, Chengdu University, Chengdu, 400001, China

## Abstract

Biosynthesis approaches for the production of higher alcohols as a source of alternative fossil fuels have garnered increasing interest recently. However, there is little information available in the literature about using undirected whole-cell mutagenesis (UWCM) *in vivo* to improve higher alcohols production. In this study, for the first time, we approached this question from two aspects: first preferentially improving the capacity of expression host, and subsequently optimizing metabolic pathways using multiple genetic mutations to shift metabolic flux toward the biosynthetic pathway of target products to convert intermediate 2-keto acid compounds into diversified C4~C5 higher alcohols using UWCM *in vivo*, with the aim of improving the production. The results demonstrated the production of higher alcohols including isobutanol, 2-methyl-1-butanol, 3-methyl-1-butanol from glucose and duckweed under simultaneous saccharification and fermentation (SSF) scheme were higher based on the two aspects compared with only the use of wild-type stain as expression host. These findings showed that the improvement via UWCM *in vivo* in the two aspects for expression host and metabolic flux can facilitate the increase of higher alcohols production before using gene editing technology. Our work demonstrates that a multi-faceted approach for the engineering of novel synthetic pathways in microorganisms for improving biofuel production is feasible.

Currently, one of the most promising approaches for replacing fossil fuels is the biological production of biofuels via genetically-modified microorganisms. Some examples, such as n-butanol and isobutanol, have been synthesized using genetically-engineered bacteria^1^,^2^,^3^,^4^,^5^. Of particular interest are longer chain hydrocarbons, such as C4~C5 higher alcohols including 3-methyl-butanol, 2-methyl-butanol, and isopentanol, which have been successfully synthesized through the use of non-synthetic *E. coli* and *Corynebacterium* based on their endogenous α-keto acid pathway^6^,^7^,^8^,^9^. These alcohols have a higher energy density close to that of gasoline, exhibit lower hygroscopicity compared to ethanol, and are currently regarded as very promising potential substitutes for fossil fuels.

One of the first challenges in the replacement of fossil fuels with biofuels derived via a biosynthesis pathway is the inefficient conversion of lignocellulosic substrate into biofuels by bacteria. To biosynthesize these C4-C5 higher alcohols by engineering bacteria such as *E. coli, Bacillus subtilis*, and *Corynebacterium glutamicum*, the fermentation substrates traditionally were pure sugar or starch without heavy metals and other impurities^9^,^10^,^11^,^12^. However, limited research has been reported using bioengineered strains to produce C4~C5 higher alcohols via fermenting hydrolysates of lignocellulose. Therefore, it is essential to investigate the capacity of bioengineered strains to directly ferment hydrolysates of lignocellulose as a more effective method of biofuel generation.

There is a growing trend to develop new, renewable, non-food plant sources as feedstock for biofuel production. Duckweed has received increasing attention as a potentially inexpensive and sustainable lignocellulose source of non-food plant biomass for producing biofuels such as ethanol and biobutanol, and has demonstrated great potential as a candidate for bioenergy feedstock^6^,^13^,^14^. However, there are no studies to date regarding the efficacy of bioengineered strains of Corynebacterium in producing other C4~C5 higher alcohols, especially 3-methyl-butanol and 2-methyl-butanol using hydrolysates of lignocellulose as a fermentation substrate under SSF scheme. Therefore, we selected duckweed as a fermentation substrate to investigate the ability of bioengineered strains of a Corynebacterium, *C. crenatium,* to produce C4~C5 higher alcohols via SSF.

Secondly, in addition to the need to overcome the inefficiency of utilizing lignocellulosic substrate to convert into biofuels, it is also essential that improvement of the expression host’s catalytic activity via introducing novel metabolic pathways with high-activity enzymes in order to maximize higher alcohols production as much as possible. One major challenge in the construction of metabolic pathways is the identification and selection of enzymes with high activity. Protein-directed engineering *in vitro* such as error-prone PCR and DNA shuffling provides a foundation for screening interesting mutant genes that are favorable for improving higher alcohols. However, there may be undesirable effects that may lead to a metabolic flux imbalance when overexpressing these mutant heterologous genes from other species via *in vitro* mutation experiment, and thus ultimately decrease the yield of higher alcohols. Unfortunately, using these methods to obtain mutant genes with high enzymatic activity while maintaining metabolic flux balance in a expression host is not always achievable.

Mutant genes with high enzymatic activity can also be obtained using UWCM *in vivo*. For example, some high yield auxotrophes, such as amino acid-producing Corynebacterium, and *Saccharomyces cerevisiae* which produces 3-methyl-1-butanol, have been obtained via perturbing whole genome using the UWCM method^15^,^16^,^17^,^18^,^19^,^20^. Using this method, multiple mutant genes are identified that have metabolic balance favorable for optimization of metabolic pathways in these auxotrophic strains. There is a dynamic equilibrium of metabolic pathways between these mutant genes and auxotrophic strains, and thus improved production. However, until now, improving higher alcohols production via overexpressing exogenous genes from the UWCM approach *in vivo* into a expression host has not been shown. Therefore, we proposed to use the method to obtain multiple gene mutations in an auxotrophic strain, and utilize those mutant genes to construct novel metabolic pathways, and then introduce those novel metabolic pathways into another expression host reach to improve production of C4~C5 higher alcohols.

Thirdly, the capability of host itself is also an important consideration for improving higher alcohols production in addition to the introduction of metabolic pathways with highly activated genes. In general, the expression hosts used to produce higher alcohols were typically wild-type strains prior to the use of gene editing techniques. Using improved auxotrophic strains from UWCM instead of wild-type strain as expression host to increase yield of specific products prior to using gene editing techniques, followed by the use of gene editing techniques to further reform the host may maximize product yield. However, this has not yet been reported in the literature.

Therefore, in this study, we aimed to improve higher alcohols production using two aspects. First, improved metabolic capability of *C. crenatium* and *S. cerevisiae* via UWCM, whereby the improved mutant *C. crenatium* was used as expression host and the mutant *S. cerevisiae* was used to extract multiple mutant genes. Addionally, these newly identified mutant genes involving in the biosynthesis of C4~C5 higher alcohols from mutant *S. cerevisiae* were used to alter the metabolic flux of the improved mutant *C. crenatium* by constructing novel metabolic pathways to improve C4~C5 higher alcohol production. We assessed the fermentation efficiency of bioengineered strains for producing higher alcohols using glucose and duckweed substrate under SSF.

## Results and Discussion

### Source of mutant genes

**Figure 1:**
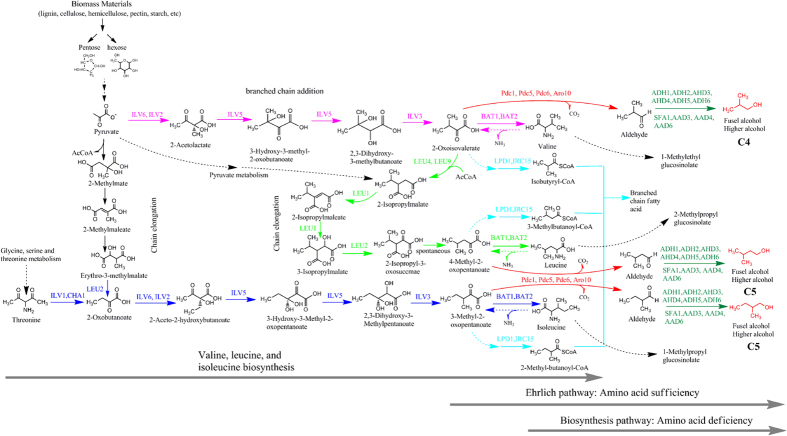
The metabolic flux distribution and relevant enzymes involved in the formation of higher alcohols, including amino acid biosynthetic pathways, decarboxylation reduction reaction, and the Ehrlich metabolic pathway (http://www.genome.jp/dbget-bin/www_bget?pathway:sce01210). *CHA1, ILV1*: L-serine/L-threonine ammonia-lyase, *IRC15*: dihydrolipoamide dehydrogenase, *LPD1*: dihydrolipoyl dehydrogenase, *LEU2*: 3-isopropylmalate dehydrogenase, *ILV1*: L-serine/L-threonine ammonia-lyase, *ILV2*: acetolactate synthase, *ILV5*: ketol-acid reductoisomerase. *ILV3*: dihydroxy-acid dehydratase, *BAT1*: branched-chain-amino-acid transaminase, *BAT2*: branched-chain amino acid aminotransferase, *LEU4*: 2-isopropylmalate synthase, *LEU1*: 3-isopropylmalate dehydratase, *IRC15*: dihydrolipoamide dehydrogenase, *LPD1*: dihydrolipoyl dehydrogenase, *CAN1*: arginine permease, *ARO10*: phenylpyruvate *PDC1*:decarboxylase pyruvate decarboxylase 1, *PDC2*: pyruvate decarboxylase 2, *PDC5*: pyruvate decarboxylase 5, *PDC6*: pyruvate decarboxylase 6, *ADH1*: alcohol dehydrogenase 1, *ADH2*: alcohol dehydrogenase 2, *ADH6*: alcohol dehydrogenase 6, *SFA1*: bifunctional alcohol dehydrogenase/S-(hydroxymethyl)glutathione dehydrogenase, *AAD3, AAD 4*: Oxidoreductases. *AAD6*: putative aryl-alcohol dehydrogenase 6, *AAD10*: putative aryl-alcohol dehydrogenase 10.

**Figure 2:**
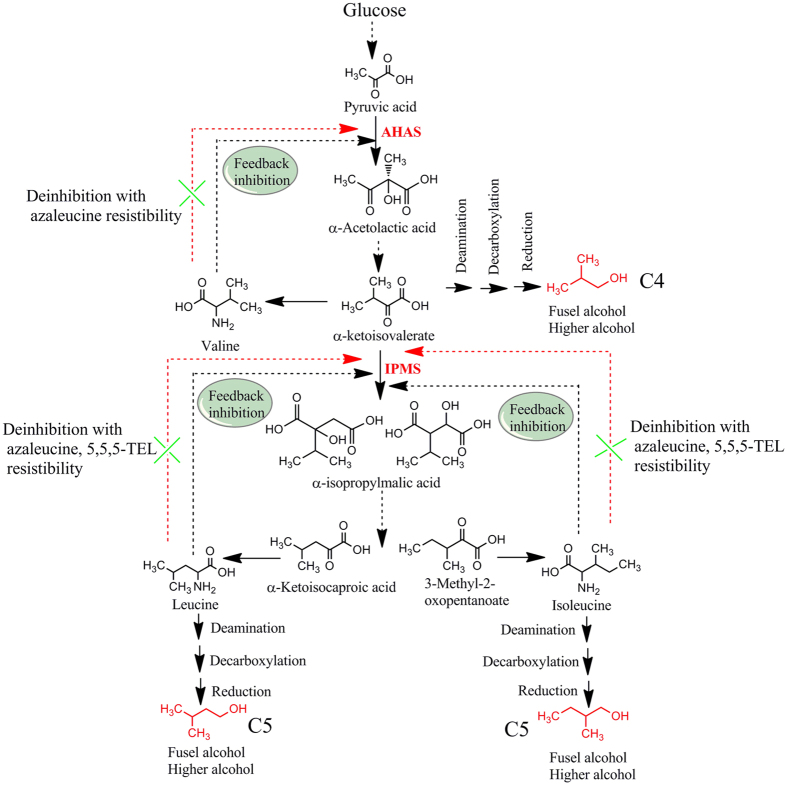
Metabolic mechanism of screening auxotrophic mutants of yeast using amino acid analogue via controlling the key enzymes based on the relationship between the formation of higher alcohols and the metabolism of amino acids. *AHAS*: acetohydroxyacid synthase, *IPMS*: α-isopropylmalate synthase.

It is well-known that the formation of higher alcohols by yeast is closely related to the metabolism of amino acids including the Ehrlich metabolic pathway^21^ and the biological synthesis pathway^22^,^23^. The metabolic flux distribution and relevant genes involved in the formation of higher alcohols are shown in Fig. 1. Previous studies have shown that metabolic perturbation derived from mutation of genes associated with the biosynthesis and degradation pathways of leucine, isoleucine, and valine in yeast can improve C4~C5 higher alcohol yield based on these two pathways^24^,^25^,^26^. Mutating acetohydroxyacid synthase (AHAS) gene responsible for transforming pyruvic acid into α-acetolactic acid can reduce the generation of the diacetyl, thereby improving the production of higher alcohols. Some reports have demonstrated the mutant strains with increased higher alcohols production can be obtained using some resistance marker such as streptomycin (SM)^27^,^28^,^29^,^30^, the arginine analogue canavanine^31^,^32^,^33^, and 2-thiazolyl-DL-alanine^34^. These studies shows that acetohydroxy acid synthase (AHAS) and α-isopropylmalate synthase (IPMS) are key genes in the generation of higher alcohols, and that feedback inhibition takes place between the two genes and their respective metabolite production^35^,^36^,^37^ (Fig. 2). Therefore, through controlling the enzymatic processes between them to block feedback inhibition, it is possible to screen out mutant leucine-deficient strains that generate high 3-methyl-1-butanol production. Auxotrophic strain can improve the production of higher alcohols as a result of the mutation of genes related to branched chain amino acid biosynthesis. For example, mutation of *ILV2* gene in valine biosynthesis can lead to improved yields of higher alcohols^35^,^36^,^37^.

**Figure 3:**
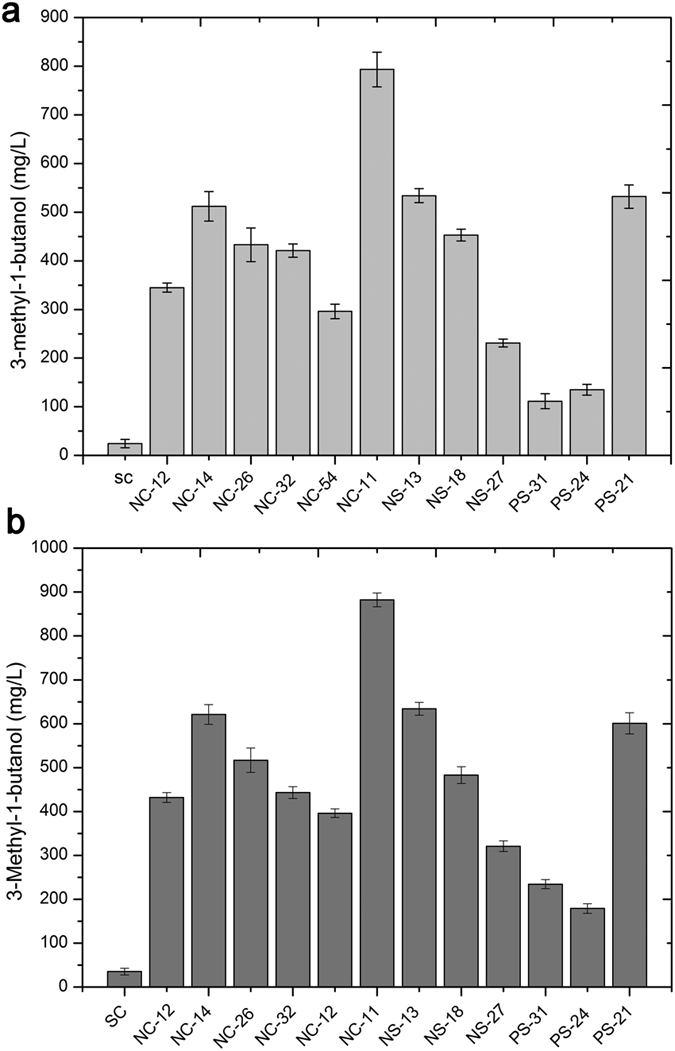
Fermentation experiments to determine the ability of each colony to produce higher alcohols. (Figure 5B) In order to verify the genetic stability of the mutant strains, glucose was used as a fermentation substrate. (Figure 5A) These positive mutant strains were then used as reinspected strains to assay the capacity for fermentation of duckweed hydrolysate. SC: Saccharomyces cerevisiae.

Learning from these previous studies, here, we used the leucine analogue 4-Aza-dl-leucine dihydrochloride (AZL) as a resistance screening compound to obtain feedback inhibition of AHAS or IPMS mutants with high 3-methyl-1-butanol production (Fig. 2). AZL has been used previously in *Bacillus subtilis, Escherichia coli*, and *Salmonella typhimurium* to identify resistant strains in which three enzymes of the leucine, isoleucine, valine metabolic pathway (isopropylmalate isomerase, isopropylmalate dehydrogenase (IPMDH), and isopropylmalate synthase (IPMS)) were inhibited^38^,^39^. These studies showed that perturbing the metabolic pathway involving AHAS regulation to obtain auxotrophic strains with some mutant genes is an effective method of generating a high yield of amino acids. And further investigated the ability of mutant strains via fermenting the hydrolysate of duckweed to identity the desirable strain with the highest 3-methyl-1-butanol. A total of 35 colonies grew after three days of incubation. The fermentative results were shown for the investigation of the capability of each colony to produce higher alcohols (Fig. 3). First, to verify the fermentative stability, these auxotrophic strains were cultured consecutively for 10 generations, and then given glucose as substrate to determine the capacity of mutant strains with stability for producing higher alcohols (Fig. 3b). Secondly, these positive auxotrophic strains with stability were used as reinspected strains to assess their capacity for fermenting duckweed hydrolysate (Fig. 3a). Compared with the original strain SC (*S. cerevisiae* AH109), there were 12 mutant strains possessed fermentative stability, and had significant higher titers of 3-methyl-1-butanol. Therein, the mutant strain NC-11 produced the highest production, approximately 25-fold as compared to the original strain. Additionally, using both glucose as well as hydrosylate demonstrated impressive capacity of the auxotrophic strain NC-11. Therefore, we selected the auxotrophic strain NC-11 as the candidate strain for the next step to survey which possible mutant genes are responsible for the increased biosynthesis of 3-methyl-1-butanol production. We hypothesized that these mutant genes were highly involved in Ehrlich metabolic pathway and/or the biological synthesis pathway, and would contribute to the improvement of higher alcohols in subsequent experiments. Thus supporting the extraction of these mutant genes in order to construct novel metabolic pathways.

### Improvement of expression host

The production of higher alcohols in synthetic microbes relies on exogenous metabolic pathways such as the decarboxylation reduction pathway that converts intermediate metabolites of the amino acid synthesis pathway or Ehrlich metabolic pathway into alcohols. Various α-keto acid intermediate compounds are converted into corresponding fatty alcohols. Therefore, increasing amino acid production can result in the accumulation of precursors, thereby providing more substrate pool for subsequent conversion, and thus improving the final total production of higher alcohols.

**Figure 4:**
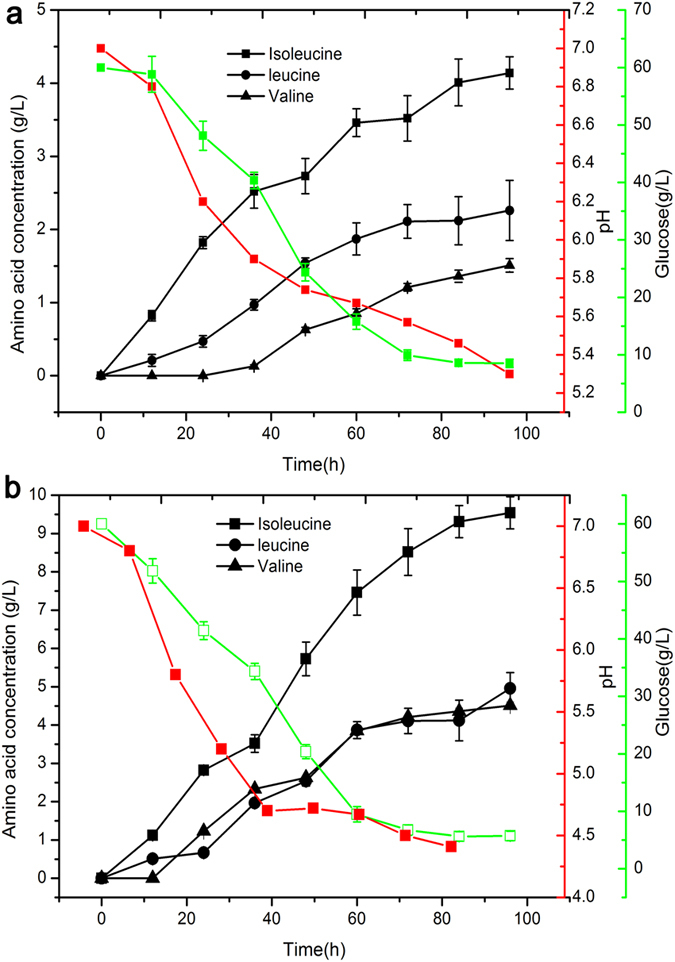
Identification experiments of the representative mutant strain *C. crenatium* MA11C to use as expression host. (Figure 6a) The original strain *C. crenatium* CICC 20135. (Figure 6b) *C. crenatium* MA11C.

Here, the representative auxotrophic strain with the highest yield of amino acid was selected from a total of 35 mutant strains after UWCM via batch fermentation using glucose. The fermentation results of each strain are shown in Fig. 4. The auxotrophic strain *C. crenatium* MA11C generated much higher levels of the amino acids isoleucine, leucine, and valine (approximate 2-fold) compared to the original strain *C. crenatium* CICC 20135 (Fig. 4a,b). In particular, the representative auxotrophic strain *C. crenatium* MA11C produced the highest isoleucine (9.54 g/L), leucine (4.96 g/L), and valine (4.51 g/L) in the least amount of time (96 h). The substrate glucose was not completely consumed by *C. crenatium* MA11C, but its consumption levels were the lowest for all investigated mutant strains. For *C. crenatium* MA11C, the residual amount of glucose was up to 5.72 g/L, compared to the *C. crenatium* CICC 20135, for which the residual glucose reached 8.52 g/L (Fig. 4a,b). The changes in pH during fermentation are shown in Fig. 4(a,b). The largest pH change observed for all investigated mutant strains during fermentation was from *C. crenatium* MA11C (from 4.42 to 7.0; Fig. 4b). At the end of fermentation, the pH values were above 4.42. The pH during fermentation by the original strain *C. crenatium* CICC 20135 stayed above 5.3 (Fig. 4a).

These results demonstrate that the residual amount of glucose for the mutant strain was lower than that of the original strain, which suggests that the mutant strains have a higher uptake of glucose. This also helps to explain the improved amino acid production from the auxotrophic strain compared to the original strain. The changes in pH values also reflect the total amino acid titer; when the final pH was lower, the amino acid titer should be higher. These results indicate that high amino acid titer is likely the result of increased precursors (α-ketoacids), and that the efficiency with which the precursor was converted into higher alcohols by *C. crenatium* MA11C may be higher than that of the original strain *C. crenatium* CICC 20135. Furthermore, the increased amino acid production of *C. crenatium* MA11C could be due to mutation of some genes in the amino acid synthesis pathway. The results via batch fermentation repeatedly demonstrated that fermentation of the auxotrophic strain is stable after being sequentially cultured for 10 generations, thus *C. crenatium* MA11C is suitable for expressing heterologous genes as expression host.

### Extraction of mutant genes

**Figure 5:**
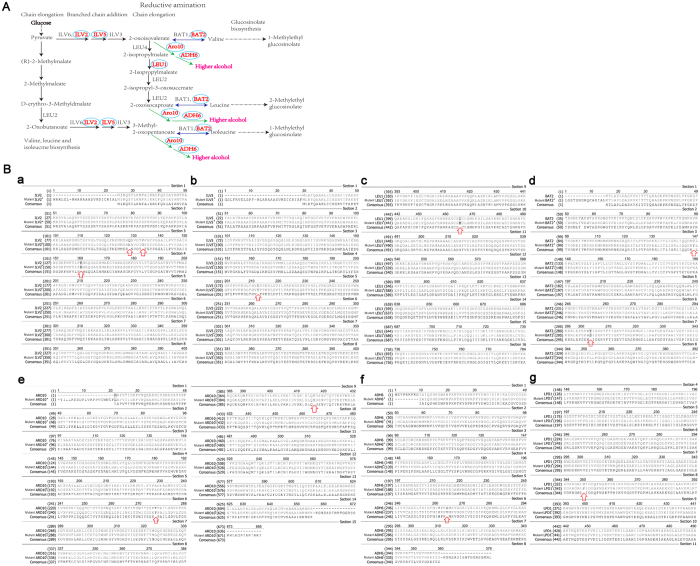
Regarding mutant genes involved in the formation of higher alcohols. (Figure 7A): the genes with red show that genes are mutated determined by our results. (Figure 7B): Sequence alignment of amino acids of mutant genes involved in biosynthesis process of higher alcohols. The gene with an asterisk indicates the gene produced mutation. The gene without asterisk indicates that the gene is native gene.

As above description, the improvement of higher alcohol production by mutant yeast strains is likely the result of mutation of genes associated with the metabolic pathways of higher alcohol intermediates, resulting in perturbation of metabolic flux via UWCM. The auxotrophic strain NC-11 was found to produce the highest yield of 3-methyl-1-butanol and has fermentation stability. Therefore, the auxotrophic strain was used for subsequent identification of potential genetic mutations. Genes related to the Ehrlich pathway and amino acid biosynthetic and degradation pathways have been implicated in the production of higher alcohols (Fig. 1), and our results showed that genes *ILV2, ILV5, BAT2, LEU1, AR010*, and *ADH6* acquired mutations (Fig. 5).

**Figure 6:**
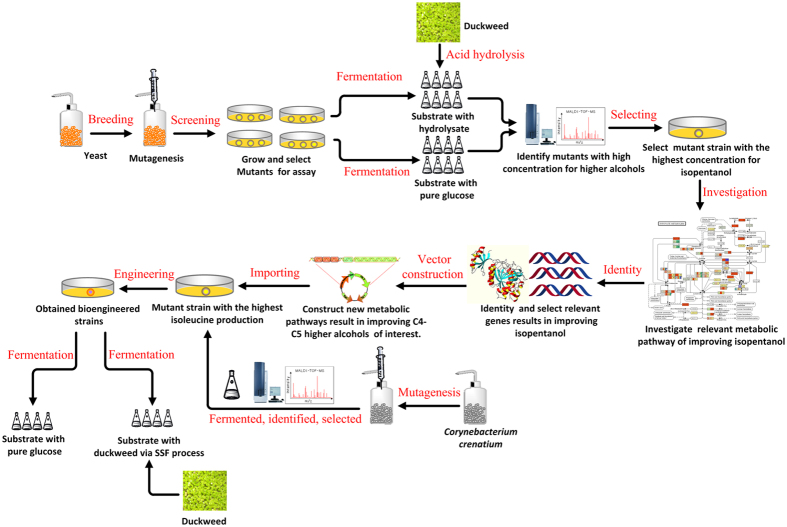
The flowsheet of experiments following the methodology illustrated for screening exogenous mutant enzymes and expression host via undirected whole-cell mutagenesis (UWCM) *in vivo* and fermentation processes.

According to this results, mutant genes ILV2*, ILV5*, and LEU1* are responsible for isoleucine, leucine, and valine biosynthetic metabolic pathways. This indicates that the metabolic flux generated a change in the synthesis of 2,3-Dihydroxy-3-methylbutanoate and 2,3-Dihydroxy-3-Methylpentanoate, which are the precursors of 2-oxoisovalerate and 3-Methyl-2-oxopentanoate, respectively (Figs 5 and 6). The LEU1* also gave rise to the metabolic flux from 2-Isopropylmalate to 3-Isopropylmalate. Mutant gene BAT2* is involved in the deamination reaction pathway, indicating that metabolic flux from valine, isobutanol, and leucine to 2-oxoisovalerate, 3-Methyl-2-oxopentanoate and 4-Methyl-2-oxopentanoate, respectively, had changed (Figs 5 and 6). These mutant genes are likely to increase accumulation of intermediate metabolites and precursors of higher alcohols such as the intermediates including isopropylmalate, 2-oxobutanoate, 2-oxoisocaproate, 2-isopropyl-2-oxosuccinate, and precursors including 3-methyl-2-oxopentanoate of 2-methyl-1-butanol, 4-methyl-2-oxopentanoate of 3-methyl-1-butanol and 2-oxoisovalerate of isobutanol. These results indicate that this genes mutation led to the abnormal metabolic flux of amino acid biosynthesis and Ehrlich pathway. Therefore, this increase in amino acid production also account for increase of 3-methyl-1-butanol production in auxotrophic strain NC-11. In addition, the gene dihydrolipoyl dehydrogenase, which is responsible for the degradation of valine, isoleucine, and leucine, and the genes *ARO10* and ADH6* involved in the decarboxylation reduction pathway, were also investigated (Figs 1 and 5). It was concluded that the reason that the strain NC-11 can produce high yields of 3-methyl-1-butanol may be due to the change of metabolic flux from these mutant genes working synergistically. These mutant genes were extracted and used to construct novel metabolic pathways.

### Higher alcohols from bioengineered strains

**Figure 7:**
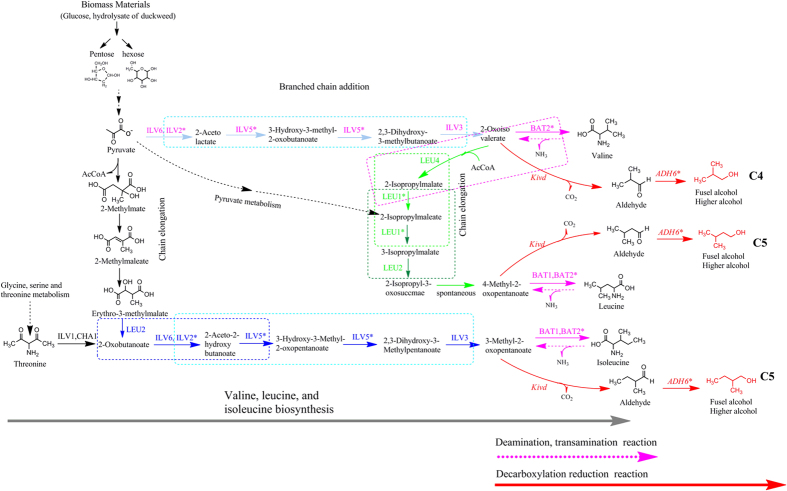
The schematic plot of designing novel metabolic pathways responsible for the improvement of higher alcohol production. Different colors rectangular frames represent different metabolic pathways. The gene with an asterisk indicats the gene produced mutation. The gene without asterisk indicats the gene is native gene. The novel metabolic pathways for accumulating precursor products is illustrated with the following pathways, labeled MEp I (*LEU2*-ILV2*-ILV5*), MepII (ILV2*-ILV5*-*ILV3*), MepIII (BAT2*), MepIV (*LEU4*-LEU1*), MepV (LEU1*-*LEU2*) and MepVI(BAT2*-*LEU4*-LEU1*), Metabolic pathway I, Ia, Ib including LEU2, ILV2*, ILV5*; Metabolic pathway II, IIa, IIb including ILV2*, ILV5*, *ILV3*; Metabolic pathway III including BAT2*; Metabolic pathway IV, IVa including LEU4, LEU1*; Metabolic pathway V, Va including LEU1*, *LEU2*; Metabolic pathway VI, VIa, VIb including BAT2*, LEU4, LEU1*. Another metabolic pathway responsible for the decarboxylation reduction reaction, and MepDRVII (*kivd*-ADH6*) Metabolic pathway VIIincluding kivd, ADH6*. *LEU2*: 3-isopropylmalate dehydrogenase, *ILV1*: L-serine/L-threonine ammonia-lyase, ILV2*: mutant acetolactate synthase, ILV5*: mutant ketol-acid reductoisomerase, *ILV3*: dihydroxy-acid dehydratase, BAT2*: branched-chain amino acid aminotransferase, *LEU4*: 2-isopropylmalate synthase, LEU1*: mutant 3-isopropylmalate dehydratase, *kivd*: alpha-ketoisovalerate decarboxylase, ADH6*: mutant alcohol dehydrogenase 6.

Engineering *C. crenatium* to express native genes from wild-type *S. cerevisiae* to produce higher alcohols have been investigated^6^. Although the yield was low, they have demonstrated the potential capability of this host to be useful in improving production of higher alcohols. In order to further improve the fermentation efficiency of *C. crenatium*, compatibility of two expression vectors in a host was first demonstrated. The gene *pBL1* from expression vector pXMJ19 was inserted into the expression vector pSVT29 to construct a new expression plasmid pTVpBL. The vectors pTVpBL and PEC-XK99E were compatible and stable in the expression host. Secondly, novel metabolic pathways were constructed using mutant genes. Thirdly, the improved mutant *C. crenatium* was used as expression host harboring novel metabolic pathways. A schematic representation of the novel metabolic pathways is shown in Fig. 7.

**Figure 8:**
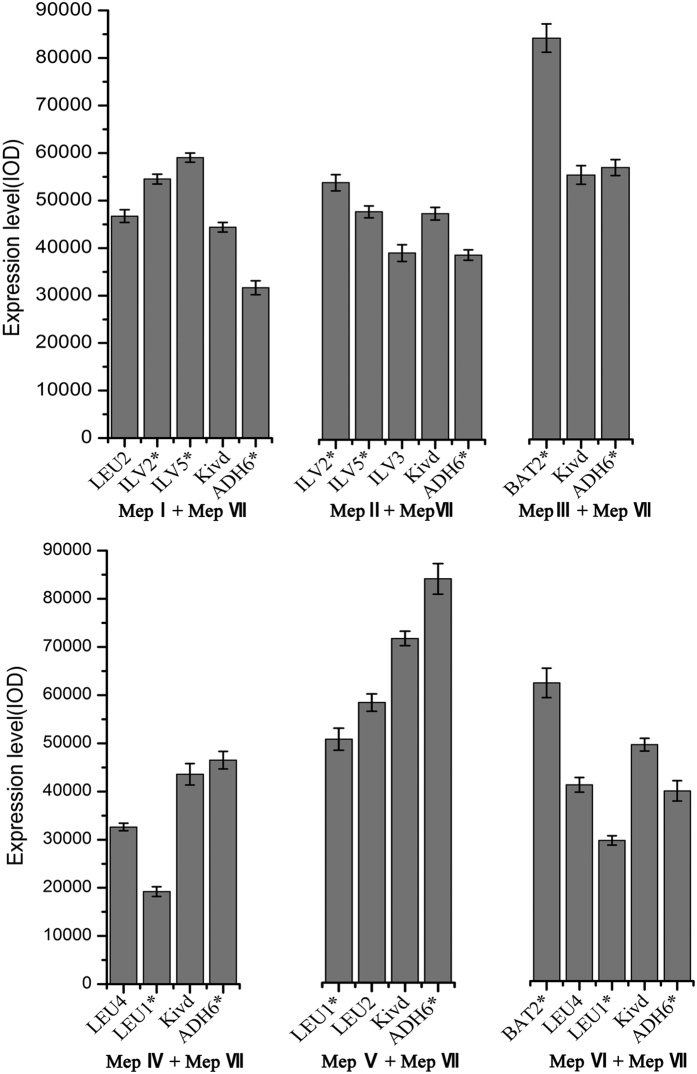
Gene expression of all recombinant genes for each metabolic pathway in the production host was analyzed using semi-quantitative RT-PCR. Semi-quantitative RT-PCR analysis: expression profiles of all recombinant genes were evaluated using Gel-Pro analyzer 4.0 software. Labeled MEp I (*LEU2*-ILV2*-ILV5*), MepII (ILV2*-ILV5*-*ILV3*), MepIII (BAT2*), MepIV (*LEU4*-LEU1*), MepV (LEU1*-*LEU2*) and MepVI(BAT2*-*LEU4*-LEU1*).

In order to guarantee the metabolic capacity of these engineered strains for producing higher alcohols, we verified whether these mutant genes involved branched-chain amino acid synthesis were overexpressed in *C. crenatium* MA11C. Expression levels of all mutant genes were confirmed by RT-PCR, and the results were further analyzed with a semi-quantitative method (Fig. 8). Our results demonstrated that the induced genes corresponding to respective metabolic pathways have been successfully expressed and that the expression level of these recombinant genes remained relatively different under similar cultivation conditions.

**Figure 9:**
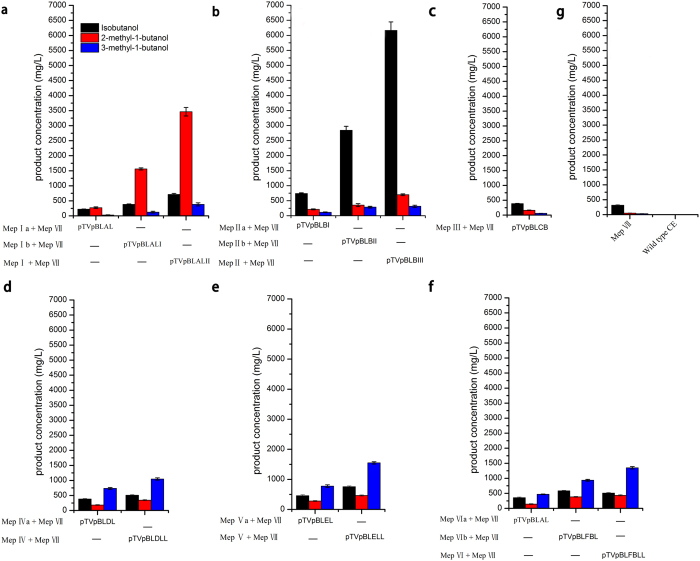
Fermentation experiment to produce higher alcohols for all constructed metabolic pathways using pure glucose. labeled MEpIa (*LEU2*), MEpIb (*LEU2*-ILV2*), MEpI (*LEU2*-ILV2*-ILV5*); MepIIa(ILV2*), MepIIb(ILV2*-ILV5*), MepII (ILV2*-ILV5*-*ILV3*); MepIII (BAT2*); MepIVa(*LEU4*), MepIV (*LEU4*-LEU1*); MepVa (LEU1*), MepV (LEU1*-*LEU2*); MepVIa ((BAT2*), MepVIb (BAT2*-*LEU4*); MepVI (BAT2*-*LEU4*-LEU1*); MepVII (*kivd*-ADH6*).

**Figure 10:**
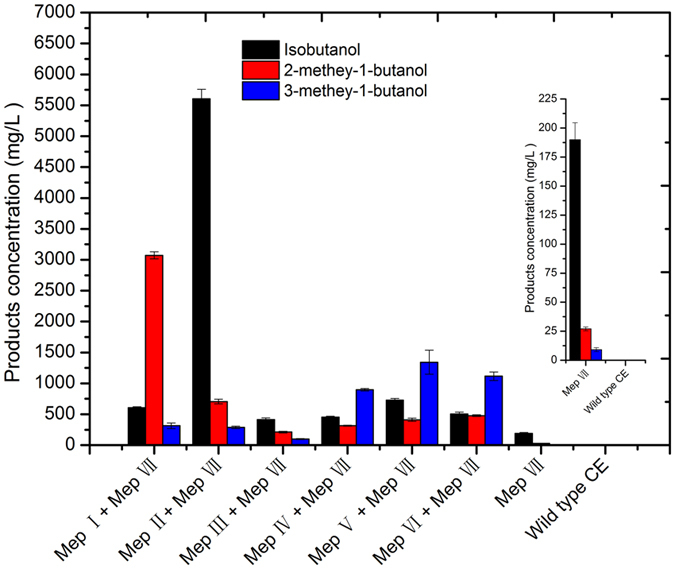
Fermentation experiment to produce higher alcohols using duckweed via SSF procedure for the most efficient mutant strain. labeled MEp I (*LEU2*-ILV2*-ILV5*), MepII (ILV2*-ILV5*-*ILV3*), MepIII (BAT2*), MepIV (*LEU4*-LEU1*), MepV (LEU1*-*LEU2*) and MepVI(BAT2*-*LEU4*-LEU1*), MepVII(*kivd*-ADH6*).

Fermentation was achieved for all novel metabolic pathways using pure glucose (Fig. 9) and duckweed via the SSF procedure (Fig. 10). To construct and overexpress two metabolic pathways Mep I and Mep VII, the highest yield of 2-methyl-1-butanol at 3476.52 mg/L was obtained using glucose as the fermentation substrate. In addition, the by-products isobutanol at 735.18 mg/L and 3-methey-1-butanol at 403.53 mg/L were obtained in 96 h (Fig. 9, Table 1). Similarly, overexpressing the two metabolic pathways simultaneously led to the highest yield of 2-methyl-1-butanol (3071.5 mg/L) and byproducts of isobutanol (605.17 mg/L) and 3-methey-1-butanol (313.73 mg/L) using the duckweed via SSF. Our results showed that a higher titer can be obtained using pure glucose compared to using duckweed. The parameters of total alcohols, productivity, and total yield were higher using glucose than those using duckweed (Table 1). However, to overexpress other metabolic pathways Mep Ia + Mep VII and Mep Ib + Mep VII, the yield was clearly lower than that of Mep Iand Mep VII when using either glucose or duckweed (Figs 9 and 10). Only 288.65 mg/L 2-methyl-1-butanol, 248.31 mg/L isobutanol, and 54.208 mg/L 3-methey-1-butanol were obtained from Mep Ia + Mep VII using glucose and approximately 1588.07 mg/L 2-methyl-1-butanol, 397.46 mg/L isobutanol, and 144.4 mg/L 3-methey-1-butanol were produced from Mep Ib + Mep VII using duckweed via SSF.

Maximum isobutanol titer 6207.15 mg/L, and productivity 76.06 mg/L/h were achieved with glucose. Using duckweed, the resulting isobutanol level was 5607.15 mg/L and productivity was 76.06 mg/L/h, which resulted from synergistically overexpressing two metabolic pathways Mep II + Mep VII. In addition, other byproducts such as 2-methyl-1-butanol (735.46 and 705.4 mg/L), and 3-methey-1-butanol (359.6 and 289.16 mg/L), can also be obtained from glucose and duckweed, respectively, and thus lead to the highest total alcohols solution 7302.21 and 6601.7 mg/L (Figs 9 and 10, Table 1), with a productivity of 76.06, 68.77 mg/L/h and total yield of 121.7 and 66.01 mg/g for glucose and duckweed, respectively (Table 1). In addition, the other metabolic pathways Mep IIa + Mep VII and Mep IIb + Mep VII can produce 773.68 and 2881.92 mg/L isobutanol, 240.8 and 393.88 mg/L 2-methyl-1-butanol, and 139.77 and 316.45 mg/L 3-methyl-1-butanol using glucose.

To summarize the results of above two novel metabolic pathways, owing to the genes ilv2* and ilv5* are closely related to the biosynthesis of isoleucine (Figs 1 and 5), it showed that the two genes catalyze not only the conversion of ketobutyrate and pyruvate, but also that of pyruvate and pyruvate to make ketomethyl valerate and ketoiso valerate, respectively (Fig. 1). The results showed the improvement of higher alcohols can be confirmed using the mutant genes ilv2* and ilv5* to construct novel metabolic pathways to constitute Mep I and Mep II. We demonstrated that increasing the pathway flux of valine allows for the accumulation of leucine, and thus improves 2-methyl-1-butanol reached to the highest yield 3476.52 mg/L. The results also showed that reinforcing the flux of pyruvate by redirecting the carbon flux via expressing ilv2*, ilv5*, and *ilv3* (Mep II) in the L-valine biosynthesis pathway can lead to significant improvement of isobutanol, reaching an increased yield 6207.15 mg/L compared to previous studies which did not utilize gene editing techniques.

Isobutanol yields were also improved by introduction of the mutant gene BAT2* via constructing metabolic pathway Mep III + Mep VII, reaching 462.76 and 412.26 mg/L isobutanol, 236.08 and 212.18 mg/L2-methyl-1-butanol and 135.02 and 99.12 mg/L from glucose and duckweed via SSF, respectively (Figs 9 and 10, Table 1). Furthermore, overexpressing the mutant gene BAT2* resulted in a higher relative proportion of C4 isobutanol than the other two C5 higher alcohols analyzed (Fig. 5). We conclude that the mutant BAT2* obtained by UWCM elicits an effect on the three branched-chain amino acids, and ultimately improved higher alcohols production. Our results revealed that mutant BAT2* supplied increased levels of precursors to valine, leucine, and isoleucine. Although *BAT2* is the only transaminase of relevance in the transamination step within the biosynthesis of the three branched-chain amino acids valine, leucine and isoleucine, the results also demonstrated a greater impact on isobutanol yield than on yields of two other alcohols. Therefore we infer that the mutant gene BAT2* specifically plays an important role in the production of isobutanol.

In a similar manner, co-expression of other metabolic pathways Mep IV + Mep VII and Mep V + Mep VII, yielded different results as presented in Table 5. Other information can be seen in Fig. 9. For example, 385.39 and 491.57 mg/L isobutanol, 176.96 and 326.4 mg/L 2-methyl-1-butanol, and 727.52 and 817.97 mg/L 3-methey-1-butanol can be generated from Mep IVa + Mep VII and Mep Va + Mep VII respectively using glucose (Fig. 9). The two metabolic pathways Mep IV and Mep V have the same mutant gene LEU1*. We noted changes in the production of higher alcohols resulting from the two metabolic pathways. The yields of all higher alcohols assayed were significantly improved by Mep V as compared to those of Mep IV. The results revealed that overexpression of the *LEU2* gene can produce much higher alcohols than overexpression of *LEU4*. This suggests that *LEU2* has stronger catalytic activity for regulating metabolic flux to enhance 3-methey-1-butanol production when simultaneously overexpressed with related genes via metabolic engineering.

To construct the metabolic pathways Mep VI + Mep VII, the highest yields of 582.02 and 502.92 mg/L isobutanol, 478.47 and 503.37 mg/L 2-methyl-1-butanol, and 1115.34 and 1415.73 mg/L 3-methyl-1-butanol were obtained from pure glucose and duckweed, respectively (Fig. 10, Table 1). Similarly, 432.58 and 660.67 mg/L isobutanol, 216.29 and 452.24 mg/L 2-methyl-1-butanol, and 530.89 and 1014.6 mg/L 3-methyl-1-butanol were generated from two metabolic pathways Mep VIa + Mep VII and Mep VIb + Mep VII using pure glucose. The increased outputs from glucose metabolism led to increases in other outcomes, including, higher total alcohol solution 2501.12 vs 2096.7 mg/L, productivity 26.05 vs 21.84 (mg/L/h), total yield 41.68 vs 20.97 (mg/g) from glucose and duckweed, respectively (Table 1). Out results showed that the yield can be improved by adding two genes *LEU4* and LEU1*.

The genes *LEU4* and *LEU2* and mutant LEU1* are involved in leucine synthesis. Specifically, *LEU4* and LEU1* are responsible for chain elongation from 2-Oxoisovalerate of the final step of L-valine synthesis to the initial step of leucine formation (Fig. 1). Therefore the catalytic activity of the LEU1* gene was confirmed by constructing MepIVand Mep, resulting in increasing leucine accumulation. The results revealed an increased enzyme level for the mutant LEU1*, which also increased metabolic flux to improve 3-methyl-1-butanol production.

These results for surveying novel metabolic pathways demonstrated that the production of higher alcohols can be greatly improved when multiple mutant genes from other species are introduced via UWCM *in vivo*, compared to the use of only native genes. The highest higher alcohols production from our experiment is higher than previous reports. For example, the highest isobutanol 151 mg/L can be obtained using *S. cerevisiae* as expression host viaby overexpression of 2-ketoisovalerate decarboxylase and valine biosynthetic enzymes^40^,^41^,^42^. The highest isobutanol, 2-methyl-1-butanol, 3-methyl-1-butanol from *C. crenatum* without improvement in cell properties and harbouring mutant genes only reach to 1264.63 mg/L, 1026.61 mg/L, 748.35 mg/L^43^. Furthermore, we showed that production of different higher alcohols can be successfully achieved via the construction of novel metabolic pathways using mutant genes from two metabolic pathways: the Ehrlich metabolic pathway and the biological synthesis pathway. In addition, these bioengineered strains of *C. crenatum* can successfully ferment duckweed via the SSF scheme to produce higher alcohols. Taking a comprehensive view of biofuel generation, such a holistic integration across multiple metabolic pathways to generate bioengineered strains that increased yields of higher alcohols is a useful approach for enhancing biofuel production using lignocellulose.

The above fermentative results indicate that the improvement of higher alcohols production is because, on the one hand, the metabolic capability of expression host itself has been improved via UWCM when used improved auxotrophic strain as expression host instead of wild-type strain. Regulating metabolic mechanisms by introducing heterogenous pathways produce generally limited production in the native expression host. The synthesizing capacity of the expression host is important but native hosts are usually not fully developed before using gene knockout methods. The improved mutant strain *C. crenatium* MA11C demonstrated a relatively high yield of precursor molecules, leading to increased higher alcohols production. The results can be indirectly demonstrated using amino acid yield because the higher alcohol intermediates and amino acids share the same precursor (Fig. 1). The *C. crenatium* MA11C produced the highest yields of isoleucine, leucine and valine, providing the first confirmation of improving higher alcohols yield. The results showed that metabolic flux can be redirected to facilitate higher alcohol production via UWCM followed by genes editing biotechnology resulting in the inactivation of competing pathways for reducing or eliminating unwanted byproducts. The amount of metabolic precursors entering competing metabolic pathways can be reduced via controlling the appropriate target products by UWCM, and thus can improve the desired production. We are continuing to work on the bioengineered strain and optimizing the process conditions to enhance higher alcohols yields using follow-up gene editing technology, to remove any bottlenecks from the pathway and further reduce by-products.

On the other hand, improvement in the production of higher alcohols may be achieved by novel metabolic pathways including multiple gene mutations. In addition to allowing the accumulation of higher alcohol precursors in host, the catalytic ability of genes is also a very important contributing factor in maximizing higher alcohol yield. Many previous studies of introduced genes generally have analyzed the effects of native genes without mutations. Limited research has been conducted in which mutant genes are generated through directed evolution technology *in vitro* to improve enzyme catalytic activity, and the target is usually a single gene. For example, the mutated *leuA* was expressed in an *E. coli* strain previously developed for increased threonine production. The yield of longer-chain alcohol 1-hexanol was improved to 146 mg/L^44^. To increase efficiency, repressive regulation of the β-oxidation cycle in the presence of glucose was avoided by using a cAMP-independent mutant (crp*) to replace the native *crp* gene^45^,^46^. However, the use of a single gene with high catalytic activity from evolution *in vitro* does not always result in a high final yield of higher alcohols as this may lead to an imbalance of metabolic flux. Furthermore, overexpression of a gene alone is generally insufficient to produce the desired final product when introduced a novel metabolic pathway. Additionally, overexpressing native genes may not always be maximally efficient for improving higher alcohol production because multiple metabolic pathways often compete for a common intermediate, leading to metabolic imbalance, which is a common problem in metabolic engineering. Therefore, UWCM *in vivo* may be an effective strategy to obtain multiple mutant genes with a synergistic effect to avoid metabolic flux imbalance.

Here, we confirmed that improvement of higher alcohols production is possible by obtaining multiple synergistic mutations associated with biosynthesis pathway by UWCM. The resulting increase in C4~C5 alcohol production by overexpressing combined multiple mutant genes might be explained by the fact that the catalytic efficiency of mutant genes is enhanced compared to native genes. Synergistic overexpression of multiple mutant genes led to a higher production of a major alcohol, and meanwhile improved the overall production of other by-products more than native genes, suggesting that the conditions in which abundant intermediate metabolites are available allow for the increase in desired end product. The results showed that a proper integration of multiple mutant genes is an attractive proposition for improving higher alcohols produce.

In conclusion, the above two aspects should be taken fully into account together to produce a high-efficiency strategy capable of improving production of higher alcohols. To overcome the obstacles to improve higher alcohols production, a synthetic multifactorial strategy is necessary that incorporates the exploitation of the synthetic capacity of the expression host with a high rate of accumulation for precursors of higher alcohols, as well as balancing the changes in metabolic flux which result from harboring multiple exogenous mutant genes. UWCM *in vivo* may be an alternative solution. We conclude that this combinatorial synthetic approach for improving upon existing pathways is feasible and will facilitate further development of processes to produce high-yield compounds from renewable resources through engineering strategies of genes editing biotechnology.

## Materials and Methods

### Experimental methodology

In this study, we sought to demonstrate the feasibility for improving C4~C5 higher biofuels production via engineering *C. crenatum* overproduction of amino acid. The methodology is illustrated in the flowchart of the experimental process (Fig. 6) including the processes of mutating wild-type *C. crenatium* and *S. cerevisiae*, identifying mutant genes, constructing novel metabolic pathways^47^,^48^, and fermentation experiment. First, we conducted the UWCM *in vivo* using the wild-type strains of *S. cerevisiae* and *C. crenatum* to generate auxotrophic strains with high metabolic capacity. Next, we identified mutant genes from auxotrophic strain of *S. cerevisiae*, and optimize metabolic flux of bioengineered strains of auxotrophic *C. crenatium* via importing a series of metabolic pathways using mutant genes. Finally, fermentation experiment were conducted using duckweed substrate under SSF. Each experiment was conducted in triplicate in a 150-mL triangular flask, unless otherwise indicated.

### Rejuvenation and propagation of microorganisms

Culture conditions for the growth, rescue, rejuvenation and seeding of the original strains are as follows. The original bacterium *C. crenatum* CICC 20135 was purchased from the China Center of Industrial Culture Collection (CICC, Beijing, China), and rejuvenated and propagated in a liquid nutrition gravy medium (LMGM) containing 5.0 g peptone, 3.0 g beef extract, 5.0 g NaCl, 20 g glucose, 1 L ddH_2_O, pH 7.0 at 30 °C in a rotary shaker for 72 h. Cultures of *Lactococcus lactis cremoris* CICC 1605 were obtained from the CICC and cultivated in liquid MRS medium containing 10 g peptone, 3.0 g beef extract, 3.0 g yeast extract, 2 g K_2_HPO_4_, 2 g citric acid diamine, 2 g sodium acetate, 20 g glucose, 0.6 g MgSO_4_·7H_2_O, 0.25 g MnSO_4_·4H_2_O, 1 L ddH_2_O, pH 6.2 at 32 °C in a rotary shaker for 72 h. Cultures of original *S. cerevisiae* AH109 were obtained from Clontech Laboratories, Inc. (Beijing, China) and propagated at 30 °C in liquid TGY medium in a rotary shaker for 72 h.

### Analysis of duckweed on basic composition

The wild duckweed (*Landoltia punctata*) was randomly gathered from the surface of wild ponds that were left uncultivated for many years in Huilong Town located in Chengdu, China. It is necessary to determine the composition of duckweed in order to provide a reference value to compare with other studies. First, fresh plants were dried at 60 °C in a drying oven (DHP9050A, Shanghai, China), then pulverized into dried powder with a pulverizer (FS100S-3, Guangzhou, China). Duckweed powder was further hydrolyzed using H_2_SO_4_ at 1% (v/v) concentration. The starch content was assessed roughly according to the total sugar content (starch content = glucose content × 0.91)^49^,^50^. The total crude protein content of duckweed was measured using the Kjeldahl Method (CP = K_j_ N × 6.25)^51^,^52^. Cellulose content was assessed roughly using spectrophotometry with the absorbance at 620 nm^53^,^54^,^55^. Processing and testing of resulting materials were performed as previously described. The content of lignin was determined using acetyl bromide according to standard methods^56^,^57^. Trace elements in duckweed were determined as follows: samples were washed with deionized water, dried at 80 °C, milled to powder, and finally digested using a wet digestion method^58^,^59^. The elemental composition in the digested solution was analyzed using atomic absorption spectrometry (Z-2300, Hitachi, Japan). The resulting components of duckweed are presented in Table 2.

### Acquisition and analysis of mutant genes

#### Mutagenesis yeast using UWCM

The original strain *S. cerevisiae* AH109 was propagated in YPDA medium after inoculation with seeding liquid in a constant temperature oscillation incubator at 30 °C, 200 rpm, until the growth solution concentration reached OD600 = 1.5. Growth solution was separated into 4 portions with equal distribution (each sample was 15 mL), and then centrifuged independently (3000 rpm for 1 minute). After centrifugation the supernatant was discarded and the cultured yeast cells were collect. Each sample was then pretreated as follows: Treatment 1 (NJ): 1.5% (v/v) methanol solution was added and kept for 10 min to shock cells; Treatment 2 (NS): cells were soaked in a solution containing 5% methanol and 0.2% polysorbate 80 for 15 min; Treatment 3 (NC): 10% sorbitol was added and incubated for 20 mins; Treatment 4 (PS), yeast cells were washed with 5 mL distilled water, then centrifuged at 3000 rpm for 5 mins, and collected. Yeast cells were resuspended in 5 mL solution containing 5% (v/v) glycerin and 10% (v/v) dimethyl sulfoxide (DMSO), and preserved in a −70 °C freezer overnight for subsequent use for further mutagenicity experiment.

Yeast cells were thawed and the pH was adjusted to 6.0 with phosphate buffer solution. The chemical mutagen 1-methyl-3-nitro-1- nitroso-guanidin (NTG) was dissolved in 1 mL acetone and added to each sample (5 mL). The final NTG concentration was adjusted to 500 μg/mL, and then cultured in a constant temperature incubator shaker at 30 °C for 15 min. Mutated cells were then collected by centrifugation and washed three times with distilled. 200 μL of resuspended mutated cells was plated on Yeast Nitrogen Base (YNB) solid medium containing 20 mg/mL 4-Aza-dl-leucine dihydrochloride (AZL) (Sigma Aldrich, USA), and then placed in 30 °C constant temperature incubator to culture approximately three days until the appearance of colonies. Finally, a fermentation experiment was conducted using fermented glucose and hydrolysate of duckweed to identify auxotrophic strains with the highest resulting C4~C5 alcohol concentration.

#### To obtain mutant yeast with high higher alcohol production

In order to identity the mutant strain with the highest efficiency for producing C4~C5 higher alcohols, we evaluated the production capacity of mutant yeast strains by conducting fermentation experiments using different fermentation substrates: pure glucose and diluted acid hydrolysates of duckweed.

For fermentation using glucose, 60 g/L glucose solution containing 5 g/L yeast extract was prepared, and a Trace Metals Mix A5 (0.222 g ZnSO4·7H_2_O g/L, 0.39 g/L Na_2_MoO_4_ · 2H_2_O, 0.079 g/L CuSO_4_ · 5H_2_O, 0.0494 g/L Co(NO_3_)_2_ · 6H_2_O, 5 g/L NaCl, 20 g/L (NH4)_2_SO_4_, 1.5 g/L KH_2_PO_4_, 0.3 g/L MgSO_4_ · 7H_2_O, 0.05 g/L FeSO_4_ · 7H_2_O, 0.01 g/L MnSO_4_ · H_2_O, 1.0 g/L CH_3_COONH) was added.

For fermentation using diluted acid duckweed hydrolysate, duckweed substrates were first pretreated using established methods of acid hydrolysis^13^. The products of hydrolysis were then fermented by mutant strains of *S. cerevisiae* AH109. The initial total glucose content of diluted acid duckweed hydrolysate was adjusted to 60 ± 3.01 g/L (before fermentation), and the pH was adjusted to 6.8 using 1% dilute phosphoric acid and 0.1% Ca(OH)_2_. The acid liquefied hydrolysates of duckweed were then used as the substrate for further fermentation verification experiments. The resulting carbohydrate composition after pretreatment at 0 h of fermentation is presented in Table 2.

The diluted acid hydrolysate of duckweed consisted of 20 mL modified M9 medium (2 g (NH_4_)_2_PO_4_, 2 g KH_2_PO_4_, 1 g K_2_HPO4, 1 g NH_4_Cl, 0.5 g NaCl, 0.5 mM MgSO_4_, 1 mM CaCl_2_, 20 mg vitamin B1, and 2 mg biotin per L of water) containing 5 g/L yeast extract, and Trace Metals Mix solution (2 g H_3_BO_3_, 2.1 g MnCl_2_·4H_2_O, 0.3 g ZnSO_4_·7H_2_O, 0.002 g MnSO_4_ 2.5 g Na_2_MoO_4_·2H_2_O, 0.05 g CuSO_4_·5H_2_O, 21.2 mg Co(NO_3_)_2_·6H_2_O, and 0.05 g FeSO_4_ per L of water) in a 150-mL triangular flask.

The substrates were fermented under aerobic conditions. Briefly, 2 mL rejuvenated seeding solution from liquid medium was inoculated into 50 mL of the substrates in a 150 mL triangular flask. The antibiotics ampicillin (100 μg/mL), chloramphenicol (35 μg/mL), and kanamycin (50 μg/mL) were added into all fermentation substrates, and kept at 30 °C to ferment with shaking at 200 rpm for 96 h in a constant-temperature oscillation incubator.

#### Identifying and extracting mutant genes

All genes were cloned into the vector pEASY-T3 cloning Vector, which contains a LacZ gene suitable for TA-cloning (TransGen Biotech Company, CT301-01, Beijing, China). The vector was propagated in Trans1-T1 Phage Resistant Chemically Competent Cells with genotype F-φ80(lacZ)ΔM15ΔlacX74hsdR(rk-, mk+ )ΔrecA1398endA1tonA (TransGen Biotech CD501-01).

All oligonucleotides were obtained from BGI (Beijing, China) with standard purification. Primer sequences were designed with vector NTI software (Informax Vector NTI Suite 11.5) and are listed in Table 3. DNA sequencing to confirm cloning products was performed by Quintara Biosciences.

Genes were amplified using their respective primers (Table 3) from the template of the mutant strain of *S. cerevisiae* AH109 that demonstrated the highest yielding alcohol production using the pEASY-Blunt Simple Cloning Kit including high fidelity polymerase (CB111-01) and TransStart FastPfu DNA Polymerase (AP221-11). The PCR products were linked to a pEASY-T3 Cloning Vector (TransGen Biotech Company) with T_4_ DNA ligase (TransGen Biotech Company), and the sequences were detected by BGI Tech. Company (Beijing, China).

### Acquisition of the expression host

#### Mutagenesis C. crenatum using UWCM

*C. crenatum* was grown up to exponential phase, the cells were centrifuged and collected at 4 °C, placed on an ice bath for 30 min, cleaned with sterile water, supplemented with 20% glycerin, and then frozen overnight. The preparation of competent cells for freezing *C. crenatum* was conducted according to the protocols mentioned in the Handbook of *Corynebacterium glutamicum*^60^. Wild-type *C. crenatum* competenct cells were collected by centrifugation at 6000 × g for 10 min at 4 °C. The supernatant was discarded, and the competent cell pellet was resuspended in 20 mL fresh LMGM medium by gentle pipetting. NTG was then added at a final concentration of 200 μg/mL. The mixture was incubated for 20 min after moderate mixing, followed by centrifugation to collect the cells. The NTG-treated cells were washed three times with PB buffer to remove residual NTG, then resuspended in 10 mL fresh LMGM medium, and incubated at 28 °C for 24 h. The NTG-treated cell suspension was diluted and plated onto LMGM solid medium. After colonies had appeared on the plate, mutants were selected to identify which auxotrophic strain had the highest isoleucine yield via fermentation experiments.

#### Identifing and obtaining the expression host

The expression host was determined to be the auxotrophic strain with the highest isoleucine titer identified via batch fermentation experiments. To prepare the fermentation inoculum for batch cultivation of mutant strains, we prepared a 100 mL TGYM medium containing 20 g/L glucose, 5 g/L yeast extract, 3 g/L ammonium acetate, 1 g/L sodium chloride, 1 g/L KH_2_PO_4_, 1 g/L K_2_HPO_4_, 0.2 g/L MgSO_4_, 0.02 g/L MnSO_4_·7H_2_O, and 0.02 g/L FeSO_4_·7H_2_O and autoclaved it at 115 °C for 20 min and cooled to 30 °C. Mutant monoclonal colonies that appeared on the plate were selected out using sterile toothpicks, transferred to sterile bottles containing TGYM medium, and cultured in a constant temperature oscillation incubator at 30 °C until the optical density (OD 600) reached 1.6.

Batch cultures were carried out for isoleucine production tests. The pH of the fermentation substrates was adjusted to 6.8 with 1% NaOH following batch culturing, which was conducted without pH control. The substrate concentration for each batch culture example was 6 0 g/L glucose, 5 g/L yeast extract, and 10 g/L peptone in a 250-mL glass anaerobic bottle (Haimen Huakai, Haimen, China) sealed with parafilm. The substrate solutions were sterilized at 115 °C for 20 min and cooled to room temperature. Subsequently, 5 mL mixture solution containing a combination of P2 trace elements (50 g/L KH_2_PO_4_, 50 g/L K_2_HPO_4_, and 220 g/L CH_3_COONH_4_), vitamins (0.1 g/L thiamin, and 0.001 g/L biotin), and minerals (20 g/L MgSO_4_·7H_2_O, 1 g/L MnSO_4_·H_2_O, 1 g/L FeSO_4_·7H_2_O, and 1 g/L NaCl) was filtered for sterilization (0.22-μm Millipore filter) and added to each bottle. The bottles were then inoculated with 0.5 mL fermentation inoculum (OD600 = 1.6), and fermented in a constant temperature oscillation incubator at 30 °C for 3 days.

#### Construction of metabolic pathways and engineering *C. crenatum*

The auxotrophic strain of *C. crenatum* CICC 20135 with the highest isoleucine titers was selected as expression host strain. All strains and plasmids used in the study are listed in Table 4. The plasmids are physically available from Addgene (http://www.addgene.org). The genes were amplified from the template of mutant strain of *S. cerevisiae* AH109 for constructing metabolic pathways using their respective primers (Table 5).

All restriction endonucleases were purchased from NEB (Shanghai, China) and T4 DNA ligase (EL0334) was supplied by MBI Fermentas (Chengdu, Beijing). All plasmids were propagated using Trans1-T1 Phage Resistant Chemically Competent Cells (TransGen Biotech Company, CD501-01, Beijing, China). The Trans1-T1 bacterial strains were cultivated in LB medium, and grown at 37 °C in a rotary shaker for 4 h. All cultures of the bioengineered strains of *E. coli* and mutant strains of *C. crenatum* CICC 20135 were then induced with 2 mM isopropyl-β-D-thiogalactoside (IPTG) and grown at 30 °C for 18 h. Antibiotics (ampicillin, 100 μg/mL; chloramphenicol, 35 μg/mL; kanamycin, 50 μg/mL) were also added when needed. The bioengineered strains were reproduced in a rotary shaker under the following conditions: *E. coli* in LB medium at 37 °C for 12 h, and *C. crenatum* in a nutrition gravy medium at 30 °C for 48 h, and moved into 4 °C to terminate the reaction.

We constructed new metabolic pathways responsible for the accumulation of higher alcohol intermediates (Fig. 7). They are designated metabolic pathway I, Ia, Ib including gene *LEU2*, mutant *ILV2*(ILV2*), and mutant *ILV5*(ILV5*); metabolic pathway II, IIa, IIb including gene mutant ILV2*, mutant ILV5*, and *ILV*3; metabolic pathway III including mutant gene *BAT2*(BAT2*); metabolic pathway IV, IVa including genes *LEU4*, and mutant *LEU1*(LEU1*); and metabolic pathway V, Va including gene mutant LEU1* and *LEU2*; metabolic pathway VI, VIa and VIb including gene mutant BAT2*, *LEU4*, and LEU1*, which were constructed using genes from auxotrophic strain *S. cerevisiae* AH109. The metabolic pathway VII responsible for oxidation-reduction reactions including genes *Kivd* and mutant *ADH6*(ADH6*) was constructed using the *Kivd* gene from *L. lactis cremoris* CICC1605 and the ADH6* gene from auxotrophic strain *S. cerevisiae* AH109.

The genes LEU2, ILV2*, and ILV5* of metabolic pathway I, Ia, and Ib were amplified with the primer pairs A1L2, A2I2, and A3I5, respectively. The genes ILV2*, ILV5* and ILV3 of metabolic pathwayII, IIa, and IIb were amplified with the primer pairs B1I2, B2I5, and B3I3, respectively. The gene BAT2* of metabolic pathway III was amplified with the primer pair CBA. The genes *LEU4* and LEU1* of metabolic pathway IV and IVa were amplified with the primer pairs D1L4 and D2L1. The genes LEU1* and *LEU2* of metabolic pathway V and Va were amplified with the primer pair E1L1 and E2L2. The genes BAT2*, *LEU4*, and LEU1* of metabolic pathway VI, VIa, and VIb were amplified with the primer pairs F1BA, F2LE4, and F3LE1 and finally the gene *pBL1*was amplified with the primer pair pBLG1. The genes *Kivd* and ADH6* of metabolic pathway VII were amplified with the primer pairs KiK1 and ADH1, respectively.

The plasmids PEC-XK99E and pSTV29 were prepared as the standard expression vectors for constructing metabolic pathways. To construct the following expression vectors pTVpBL, PEC-KA6, PTVpBLAL, pTVpBLALI, pTVpBLALII, pTVpBLBI, pTVpBLBII, pTVpBLBIII, pTVpBLCB, pTVpBLDL, pTVpBLDLL, pTVpBLEL, pTVpBLELL, pTVpBLFB, pTVpBLFBL, and pTVpBLFBLL, the construction process was manipulated using the following instructions. First, the gene *pBL1* from the standard expression vector pXMJ19 was inserted into the standard expression vector pSVT29 using restriction enzymes *Sac*II and *Cla*I to construct a new expression plasmid pTVpBL. The in-series genes Kivd and ADH6* of metabolic pathway VII were inserted into standard expression vector PEC-XK99E using restriction enzymes *Pst*I, *Xba*I, and *Kpn*I to construct expression plasmid PEC-KA6. The in-series genes LEU2-ILV2*-ILV5* of metabolic pathway I, Ia, and Ib were inserted into the new vector pTVpBL using restriction enzymes *Sph*I, *Sal*I, *Bam*HI, *Sac*I, and T4 ligase to construct plasmids pTVpBLAL, pTVpBLALI, and pTVpBLALII including different expression genes. The in-series genes ILV2*-ILV5*-*ILV*3 of metabolic pathway II, IIa, and IIb were inserted into vector pTVpBL using restriction enzymes *Pst*I, *Sal*I, *Bam*HI, *Sac*I and T4 ligase to construct expression plasmids pTVpBLBI, pTVpBLBII, and pTVpBLBIII responsible for expressing different genes, respectively. The gene BAT2* of metabolic pathway III was inserted into the vector pTVpBL using restriction enzymes *Sbf*I, *Bam*HI, and T4 ligase to construct plasmid pTVpBLCB. The in-series genes *LEU4*-LEU1* of metabolic pathway IV and IVa were inserted into the vector pTVpBL using restriction enzymes *Sbf*I, *Xma*I, *Sac*I, and T4 ligase to construct plasmids pTVpBLDL and pTVpBLDLL in charge of different catalytic reactions. The in-series genes *LEU*1*-*LEU2* of metabolic pathway V and Va were inserted into the vector pTVpBL using restriction enzymes *Sbf*I, *Bam*HI, *Sph*I, and T4 ligase to construct plasmids pTVpBLEL and pTVpBLELL. The in-series genes BAT2*, *LEU4*, and LEU1* of metabolic pathway VI, VIa, and VIb were inserted into the vector pTVpBL using restriction enzymes *Sbf*I, *Bam*HI, *Xma*I, *Sac*I, *Sph*I, and T4 ligase to construct plasmids pTVpBLFB, pTVpBLFBL, and pTVpBLFBLL, which are responsible for different metabolic reactions. The ribosome binding site (RBS) sequence was inserted into 6−8 nucleotides upstream of each structural gene to facilitate mRNA translation.

Competent cells of mutant strain *C. crenatum* were prepared as previously described in the Handbook of *C. glutamicum*^60^. All construction plasmids were introduced into mutant strains of *C. crenatum* using electroporation^61^,^62^. Electroporation was conducted according to the following manipulative conditions 100 Ω, 50 μF, 2.2 kV, and 8 ms using the Gene Pulser Xcell Microbial System165-2662 (BIO-RAD, Chengdu)..

### Gene expression analysis

Gene expression of all recombinant genes for each metabolic pathway in the production host was analyzed using semi-quantitative RT-PCR. RNA extraction and cDNA synthesis were performed according to the operation manual of TransScript First-Strand cDNA Synthesis SuperMix kit of the manufacturer (TransGen Biotech, Beijing, China). For semi-quantitative RT-PCR analysis, expression profiles of all recombinant genes were evaluated based on semi-quantified analysis using Gel-Pro analyzer 4.0 software (Media Cybernetics, Silver Spring, MD, USA), and the calculated relative expression values for all exogenous genes were calculated using integral optical density (IOD).

### Batch fermentation using bioengineered strains

#### Fermentation using glucose

Glucose (60 g/L) was used as a fermentation substrate. Glucose was sterilized at 115 °C for 20 h, then cooled to room temperature for use in fermentation assays. Batch fermentation cultures were carried out under aerobic conditions, and the pH of glucose before fermentation was automatically maintained at 6.5 by a pH controller for 24 h (PHC-2201; Able, Tokyo, Japan). The fermentation processes were conducted in 250 mL glass anaerobic bottles (Haimen Huakai experiment glass instrument Co., Ltd, Haimen, China) sealed with sealing film. After addition of 1 g yeast extract and 2 g peptone to each bottle, 5 mL of a combination of P2 trace elements mixture solution (50 g/L KH_2_PO_4_, 50 g/L K_2_HPO_4_, and 220 g/L CH_3_COONH_4_), vitamins (0.1 g/L para-aminobenzoic acid, 0.1 g/L thiamin, and 0.001 g/L biotin), and minerals (20 g/L (NH_4_)_2_SO_4_, 1.5 g/L KH_2_PO_4_, 0.3 g/L MgSO_4_ ∙ 7H_2_O, 0.05 g/L FeSO_4_ ∙ 7H_2_O, 0.01 g/L MnSO_4_ ∙ H_2_O, 1.0 g/L CH_3_COONH and 1 g/L NaCl) were filter sterilized (Millipore filter; 0.22 μm) and added to fermentation solutions in bottle. In addition, 2 mM IPTG and antibiotics (ampicillin, 100 μg/mL; chloramphenicol, 35 μg/mL; kanamycin, 50 μg/mL) were also added. The bottles were then inoculated with 0.5 mL fermentation inoculum (OD600 = 1.6), and fermented in constant temperature oscillation incubator at 30 °C for 3 days.

#### Fermentation using duckweed under SSF

For the enzymatic hydrolysis method, the fermentation substrate in duckweed trials was hydrolysate in 20 mL modified M9 medium (2 g (NH4)2PO4, 2 g KH2PO4, 1 g K_2_HPO_4_, 1 g NH_4_Cl, 0.5 g NaCl, 0.5 mM MgSO_4_, 1 mM CaCl_2_, 20 mg vitamin B1, and 2 mg biotin per L of water) containing 5 g/L yeast extract. The pH of the slurry of duckweed was adjusted to 6.0 with 1% H_3_PO_4_, and 0.2 mg/g α-amylase (120 KUN/g) was added, then hydrolyzed at 50 °C for 6 h. Subsequently, 0.2 mg/g β-amylase was added, and saccharified at 45 °C for 10 h. After that, 0.2 mg/g cellulase (500000 U/g, where the enzyme activity (U/g) is defined as follows: CMCA = 1 g enzyme powder decomposes the substrate CMC-Na to produce 1 mg glucose with treatment at 50 °C and pH 4.8 for 1 h; Thinkly, China) and 0.2 mg/g Optimash BG (containing 5.4 U β-glucosidase activity and 1.9 U β-xylosidase activity; Genencor, USA) were added to the solution. The reaction mixture was then buffered with 50 mM phosphate buffer at pH 5.0 and incubated on a rotary shaker (HZQ-X500; Yiheng, Shanghai, China) at 220 rpm for 12 h at 50 °C.

For the SSF procedure, the fermentation procedure was simultaneously conducted alongside enzymatic hydrolysis in a 150-mL triangular flask. Supplementary Trace Metals Mix solution (2 g H_3_BO_3_, 2.1 g MnCl_2_ ∙ 4H_2_O, 0.3 g ZnSO_4_ ∙ 7H_2_O, 0.002 g MnSO_4_, 2.5 g Na_2_MoO_4_ ∙ 2H_2_O, 0.05 g CuSO_4_ ∙ 5H_2_O, 21.2 mg Co(NO_3_)_2_ ∙ 6H_2_O, and 0.05 g FeSO_4_ per L of water) and the substrates were fermented under aerobic conditions. 2 mL rejuvenated seeding solution of bioengineered strains from liquid medium was inoculated into 50 mL of the fermentation substrate. All cultures were induced with 2 mM IPTG, kanamycin, and chloramphenicol. The pH of the fermentation substrate after enzymatic hydrolysis was automatically maintained at 6.5, with continued fermentation at 30 °C with shaking at 200 rpm for 96 h in a constant-temperature oscillation incubator.

### Analysis production and data statistics

Alcohol compounds were measured with a model 6890 gas chromatograph (GC) equipped with a flame ionization detector (Agilent Technologies, Santa Clara, CA, USA) with a model 7673 A automatic injector, sampler, and controller (Hewlett-Packard). Alcohol compounds were separated out using a ZB-WAX capillary column (30 m, 0.25 mm inside diameter, 0.25 μm film thickness; Phenomenex Inc., PA, USA). The GC oven temperature was held initially at 40 °C for 5 min, then raised stepwise, by 15 °C/min, until it reached 150 °C. It was then raised by 50 °C/min up to 250 °C, and held for 4 min. Helium was used as carrier gas, with an inlet pressure of 9.3 lb/in ref. 2. The injector and detector were maintained at 220 °C. A 1-μL volume of supernatant from the culture broth was injected in split-injection mode at a 1:30 split ratio. For other secreted metabolites, the constituent compounds (20 μL) were detected with an Agilent 1100 high-performance liquid chromatography system equipped with an auto-sampler and a Bio-Rad (Hercules, CA: carbohydrate analysis column Aminex HPX-87P Column 300 × 7.8 mm catalog 125-0098 serial 426070) (5 mM H_2_SO_4_, 0.6 mL/min; column temperature at 65 °C). Glucose was detected with an ELSD 2000 CSC detector, while organic acids were detected using a photodiode array detector at 210 nm. Concentrations were determined using extrapolation from standard curves. Amino acid and other organic acid production were determined with a DIONEX UltiMate 3000 liquid chromatograph in a column packed with Aminex HPX-87H and 0.05 mM H_2_SO_4_ on Chromosorb WAW. Chromatography was conducted at an injector temperature of 175 °C, detector temperature of 180 °C, and oven temperature of 125 °C.

For each experiment, all results were repeated for three times, we calculated the mean response variables and their standard deviations (SD), unless otherwise indicated. Comparisons of variable(s) were made with Student’s t-test; values of P < 0.05 were considered to indicate statistically significant differences. Tukey’s honest significant difference test was used when the null hypothesis was rejected (P < 0.05). Statistical analyses were conducted using the software program SPSS 21.0 (IBM, USA).

## Additional Information

**How to cite this article**: Su, H. *et al*. Metabolic engineering of *Corynebacterium crenatium* for enhancing production of higher alcohols. *Sci. Rep.* **6**, 39543; doi: 10.1038/srep39543 (2016).

**Publisher's note:** Springer Nature remains neutral with regard to jurisdictional claims in published maps and institutional affiliations.

## Figures and Tables

**Table 1:** The highest production and parameters corresponding to respective metabolic pathway.

Parameters	Mep I + Mep VII	Mep II + Mep VII	Mep III + Mep VII	Mep IV + Mep VII	Mep V + Mep VII	Mep VI + Mep VII	Mep VII
The highest isobutanol (mg/L)	735.18_*p*_^*a*^/615.03_*d*_^*b*^	6207.15_*p*_^*a*^/5607.15_*d*_^*b*^	462.76_p_^*a*^/412.26_d_^b^	503.37_*p*_^*a*^/453.27_*d*_^*b*^	798.31_*p*_^*a*^/728.31_*d*_^*b*^	582.02_*p*_^*a*^/502.92_*d*_^*b*^	296.9_*p*_^*a*^/189.94_*d*_^*b*^
The highest 2-methyl-1-butanol (mg/L)	3476.52_*p*_^*a*^/2974.52_*d*_^*b*^	735.46_*p*_^*a*^/705.4_*d*_^*b*^	236.08_*p*_^*a*^/212.18_*d*_^*b*^	346.06 _*p*_^*a*^/316.26_*d*_^*b*^	491.57_*p*_^*a*^/411.57_*d*_^*b*^	503.37_*p*_^*a*^/478.47_*d*_^*b*^	36.02_*p*_^*a*^/27.05_*d*_^*b*^
The highest 3-methyl-1-butanol (mg/L)	403.53_*p*_^*a*^/313.73_*d*_^*b*^	359.6_*p*_^*a*^/289.16_*d*_^*b*^	135.02_*p*_^*a*^/99.12_*d*_^*b*^	1034.26_*p*_^*a*^/897.26_*d*_^*b*^	1573.03_*p*_^*a*^/1343.13_*d*_^*b*^	1415.73_*p*_^*a*^/1115.3_*d*_^*b*^	14.28_*p*_^*a*^/9.18_*d*_^*b*^
Total alcohols (mg/L)	4615.23_*p*_/3903.3_*d*_	7302.21_*p*_/6601.7_*d*_	833.86_*p*_/723.56_*d*_	1883.69_*p*_/1666.8_*d*_	2853.91_*p*_/2483.0_*d*_	2501.12_*p*_/2096.7_*d*_	347.2_*p*_/226.2_*d*_
Productivity(mg/L/h)	48.07_*p*_/40.66_*d*_	76.06_*p*_/68.77_*d*_	8.69_*p*_/7.53_*d*_	19.62_*p*_/17.36_*d*_	29.72_*p*_/25.86_*d*_	26.05_*p*_/21.84_*d*_	3.62_*p*_/2.36_*d*_
Total yield (mg/g)	76.92_*p*_/39.03_*d*_	121.7_*p*_/66.01_*d*_	13.89 _*p*_/7.23_*d*_	31.39_*p*_/16.67_*d*_	47.56_*p*_/24.83_*d*_	41.68/20.97_*d*_	5.79_*p*_/2.26_*d*_
Fermentation time (hours)	96	96	96	96	96	96	96

^*a*^: the highest yield from pure glucose; ^*b*^: the highest yield from duckweed via SSF approach.

_*p*_: the parameter was obtained based on pure glucose; _*d*_: the parameter was obtained based on duckweed substrate.

Total yield (mg/g): total alcohols production divided by gross weight of duckweed.

Productivity (mg/L.h): Total alcohols solvents divided by fermentation time.

**Table 2:** The main components of wild duckweed *Landoltia punctate* (before fermentation: 0 h).

**Main composition of duckweed (%)**					
Sample	Water	Cellulose	Protein	Starch	Lignin
Dried duckweed	—	40.08 ± 1.37	22.53 ± 1.13	27.89 ± 1.21	2.38 ± 0.31
Fresh duckweed	83 ± 5.32	4.34 ± 0.11	2.89 ± 0.24	3.74 ± 0.83	0.27 ± 0.063
**Hydrolysis product composition via acid pretreatment**					
Pretreatment Sample	Glucose^a^	xylose^a^	Galactose^a^	Fructose^a^	Arabinose^a^
Dried duckweed	4.12 ± 0.35	2.13 ± 0.27	0.31 ± 0.11	1.14 ± 0.08	1.21 ± 0.17
Fresh duckweed	1.62 ± 0.31	1.12 ± 0.35	0.12 ± 0.043	0.42 ± 0.046	0.24 ± 0.15
**Metallic element composition of duckweed (μg/g)**					
Composition	Concentration	Composition	Concentration	Composition	Concentration
Mg	5.24 ± 0.45	Cr	3.07 ± 0.53	Zn	301.11 ± 5.25
P	343.03 ± 3.23	Mn	501.13 ± 5.47	Pb	31.27 ± 1.74
K	1167.89 ± 4.32	Cd	5.11 ± 0.38	Al	401.32 ± 6.13
Ca	154.25 ± 3.13	Fe	802.52 ± 4.27		

^a^Content (g) of various main composition of duckweed measured in 10 g pretreatment samples.

**Table 3:** Detected genes and primers used in this study.

Genes to be detected	Primer names	Base sequence (5′ to 3′)
LEU2	leu2-1	ATGTCTGCCCCTAAGAAGAT
	leu2-2	TTAAGCAAGGATTTTCTTAA
ILV1	ilv1-1	ATGTCAGCTACTCTACTAA
	ilv1-2	GCGGCTTAATATTTCAAGA
ILV2	ilv2-1	ATGATCAGACAATCTACGCTAA
	ilv2-2	TCAGTGCTTACCGCCTGTAC
ILV5	ilv5-1	ATGTTGAGAACTCAAGCCGC
	ilv5-2	TTATTGGTTTTCTGGTCTCAAC
ILV3	ilv3-1	ATGGGCTTGTTAACGAAAGT
	ilv3-2	TCAAGCATCTAAAACACAACC
BAT1	BAT1-1	ATGTTGCAGAGACATTCCTT
	BAT1-2	TTAGTTCAAGTCGGCAACAG
BAT2	BAT2-1	ATGACCTTGGCACCCCTAGAC
	BAT2-2	TCAGTTCAAATCAGTAACAACCC
LEU4	LEU4-1	ATGGTTAAAGAGAGTATTATTG
	LEU4-2	TTATGCAGAGCCAGATGCCG
LEU1	LEU1-1	ATGGTTTACACTCCATCCAA
	LEU1-2	CTACCAATCCTGGTGGACTT
LEU2	LEU2-1	ATGTCTGCCCCTAAGAAGAT
	LEU2-2	TTAAGCAAGGATTTTCTTAA
IRC15	IRC15-2	CTATTCCCGGACATGTACGC
	IRC15-1	ATGGGAGGTGAAGACGAAAT
LPD1	LPD1-1	ATGTTAAGAATCAGATCACTCC
	LPD1-2	TCAACAATGAATAGCTTTATC
CAN1	CAN1-1	ATGACAAATTCAAAAGAAGACGCC
	CAN1-2	CTATGCTACAACATTCCAAAATTTG
ARO10	ARO10-1	ATGGCACCTGTTACAATTGAAAA
	ARO10-2	CTATTTTTTATTTCTTTTAAGTGCC
PDC1	PDC1-1	ATGTCTGAAATTACTTTGGG
	PDC1-2	TTATTGCTTAGCGTTGGTAG
PDC5	PDC5-1	ATGTCTGAAATAACCTTAGG
	PDC5-2	TTATTGTTTAGCGTTAGTAGC
PDC6	PDC6-1	ATGTCTGAAATTACTCTTGG
	PDC6-2	TTATTGTTTGGCATTTGTAG
ADH1	ADH1-1	ATGTCTATCCCAGAAACTCA
	ADH1-2	TTATTTAGAAGTGTCAACAACG
ADH2	ADH2-1	ATGTCTATTCCAGAAACTCA
	ADH2-2	TTATTTAGAAGTGTCAACAACG
ADH6	ADH6-1	ATGTCTTATCCTGAGAAATTT
	ADH6-2	CTAGTCTGAAAATTCTTTGTCG
SFA1	SFA1-1	ATGTCCGCCGCTACTGTTGG
	SFA1-2	CTATTTTATTTCATCAGACTTC
AAD6	AAD6-1	ATGGCTGATTTATTTGCTCC
	AAD6-2	TCAACAGGTTCCATTTACCT
AAD10	AAD10-1	ATGGCATCAAGAAAACTGCGT
	AAD10-2	CTAATCTTCGAAGCTAATCTTGG

“1”: Sense strand; “2”: Antisense strand.

**Table 4:** The bacterial strains and vectors used in the bioengineering of bacteria to produce higher alcohols.

Metabolic pathways	Strain or plasmid	Relevant genotype^*a*^	Source
	Strains		
	DH5α	F^−^, φ 80d*lacZ* ΔM15, Δ(*lacZYA*-*argF*)U169, *deoR, recA1, endA1, hsdR17*(*rK*^−^, *mK*^+^), *phoA, supE44, λ*^−,^*thi*^*−1*^, *gyrA96, relA1*	Takara: 9057
	*C. crenatum*	*ompT, hsdSB (rB*^−^*mB*^−^), *gal, dcm*	CICC 20153
	Plasmids		
	pSTV29	pACYC184 ori; Cm^r^; PLlacO^−1^: MCS	Takara: 3332
	pTVpBL	pBL1ori; Cm^r^; PLlacO^−1^: MCS	This study
	PEC-XK99E	pGA1 Km^r^ pTrc99A MCS P-trc, lacI^q^	From CAS
	pXMJ19	pBL1ori; Km^r^; MCS P-trc, lacI^q^	From CAS
Mep VII	PEC-KA6	pGA1 Km^r^ pTrc99A MCS P-trc, lacI^q^: *Kivd*(LL)-ADH6*(NC-11)	This study
Mep Ia	pTVpBLAL	pBL1ori; Cm^r^; PLlacO^−1^:MCS:*LEU2*(NC-11)	This study
Mep Ib	pTVpBLALI	pBL1ori; Cm^r^; PLlacO^−1^: *LEU2*(NC-11)-ILV2*(NC-11)	This study
Mep I	pTVpBLALII	pBL1ori; Cm^r^; PLlacO^−1^: *LEU2*(NC-11)-ILV2*-ILV5*(NC-11)	This study
Mep IIa	pTVpBLBI	pBL1ori; Cm^r^; PLlacO^−1^: ILV2*(NC-11)	This study
Mep IIb	pTVpBLBII	pBL1ori; Cm^r^; PLlacO^−1^: ILV2*(NC-11)-ILV5*(NC-11)	This study
Mep II	pTVpBLBIII	pBL1ori; Cm^r^; PLlacO^−1^: ILV2*(NC-11)-ILV5*(NC-11)-*ILV3*(NC-11)	This study
Mep III	pTVpBLCB	pBL1ori; Cm^r^; PLlacO^−1^: BAT2*(NC-11)	This study
Mep IVa	pTVpBLDL	pBL1ori; Cm^r^; PLlacO^−1^: *LEU4*(NC-11)	This study
Mep IV	pTVpBLDLL	pBL1ori; Cm^r^; PLlacO^−1^:*LEU4*(NC-11)-LEU1*(NC-11)	This study
Mep Va	pTVpBLEL	pBL1ori; Cmr; PLlacO^−1^: LEU1*(NC-11)	This study
Mep V	pTVpBLELL	pBL1ori; Cm^r^; PLlacO^−1^: LEU1*- *LEU2* (NC-11)	This study
Mep VIa	pTVpBLFB	pBL1ori; Cm^r^; PLlacO^−1^: BAT2*(NC-11)	This study
Mep VIb	pTVpBLFBL	pBL1ori; Cmr; PLlacO^−1^: BAT2*(NC-11)-*LEU4*(NC-11)	This study
Mep VI	pTVpBLFBLL	pBL1ori; Cm^r^; PLlacO^−1^: BAT2*(NC-11)-*LEU4*(NC-11)- LEU1*(NC-11)	This study

NC-11: *S. cerevisiae* NC-11.

**Table 5:** Primers used in amplification genes for constructing metabolic pathways in this study.

Number	Primers name	Primer 5′-3′
A1L2	LEU2-S	ACATGCATGCGGATGTCTGCCCCTAAGAAGAT
	LEU2-AS	ACGCGTCGACTTAAGCAAGGATTTTCTTAACTTC
A2I2	ILV2*-S	ACGCGTCGACAAGGAGCCAGATGATCAGACAATCTACGCT
	ILV2*-AS	CGCGGATCCTCAGTGCTTACCGCCTGTAC
A3I5	ILV5*-S	CGCGGATCCAAGGAGGCCTCATGTTGAGAACTCAAGCCGC
	ILV5*-AS	GCGAGCTCTTATTGGTTTTCTGGTCTCAACTT
B1I2	ILV2*-S	AAAACTGCAGATGATCAGACAATCTACGCT
	ILV2*-AS	ACGCGTCGACTCAGTGCTTACCGCCTGTAC
B2I5	ILV5*-S	ACAGGTCGACAAGGAGGTCATGTTGAGAACTCAAGCCGC
	ILV5*-AS	CGCGGATCCTTATTGGTTTTCTGGTCTCAA
B3I3	ILV3-S	CGCGGATCCAAGGAGCTGCATGGGCTTGTTAACGAAAGT
	ILV3-AS	GCGAGCTCTCAAGCATCTAAAACACAACC
CBA	BAT2*-S	CGGCCTGCAGGATGACCTTGGCACCCCTAGA
	BAT2*-AS	CGCGGATCCTCAGTTCAAATCAGTAACAA
E1L1	leu1*-2as	CGCGGATCCAAAGGAGGCCGCATGGTTTACACTCCATCCAAGG
	leu1*-2s	TCCCCCCGGGCTACCAATCCTGGTGGACTTT
E2L2	leu2-2s	TCCCCCCGGGAAAGGAGGCCGCATGTCTGCCCCTAAGAAGATC
	leu2-2as	GCGAGCTCTTAAGCAAGGATTTTCTTAAC
D1L4	leu4-2as	CGGCCTGCAGGATGGTTAAAGAGAGTATTAT
	leu4-2s	TCCCCCCGGGTTATGCAGAGCCAGATGCCG
D2L1	LEU1*-S	TCCCCCCGGGAAGGAGACTAATGGTTTACACTCCATCCAA
	LEU1*-AS	GCGAGCTCCTACCAATCCTGGTGGACTTT
F1BA	BAT2*-S	CGGCCTGCAGGATGACCTTGGCACCCCTAGA
	BAT2*-AS	CGCGGATCCTCAGTTCAAATCAGTAACAA
F2LE4	leu4-2as	CGGCCTGCAGGATGGTTAAAGAGAGTATTAT
	leu4-2s	TCCCCCCGGGTTATGCAGAGCCAGATGCCG
F3LE1	leu1*-2s	CGGCCTGCAGGATGGTTTACACTCCATCCAA
	leu1*-2as	CGCGGATCCCTACCAATCCTGGTGGACTT
KiK1	Kivd-s	AAAACTGCAGATGTATACAGTAGGAGATTACCT
	Kivd-as	GCTCTAGATTATGATTTATTTTGTTCAGC
ADH1	ADH6*-s	GCTCTAGAAGGAAACTCAATGTCTATTCCAGAAACTCAA
	ADH6*-as	CGGGGTACCTTATTTAGAAGTGTCAACAACG
pBLG1	pBL1-as	TCCCCGCGGATTCGGGGTCGTTCACTGGT
	pBL1-s	CCATCGATAACAACAAGACCCATCATAG

s = sense and as = antisense.
